# Inspiring IDEA: Girls on the Run’s developmental approach to and assessment of inclusion, diversity, equity, and access programming

**DOI:** 10.3389/fpsyg.2023.1128680

**Published:** 2023-05-12

**Authors:** Maureen R. Weiss, Lindsay E. Kipp, Allison Riley

**Affiliations:** ^1^School of Kinesiology, University of Minnesota, Minneapolis, MN, United States; ^2^Department of Health and Human Performance, Texas State University, San Marcos, TX, United States; ^3^Girls on the Run International, Charlotte, NC, United States

**Keywords:** out-of-school-time, positive youth development, social-emotional learning, physical activity, mixed methods, diversity, equity, inclusion

## Abstract

**Introduction:**

Evaluation studies of positive youth development (PYD) programs show promising impact on children’s psychosocial and behavioral outcomes, but less is known about how programming affects youth of varying racial, ethnic, and cultural identities. *Girls on the Run*, a physical activity-based PYD program, has developed curricula and coach training with a lens toward inclusion, diversity, equity, and access (IDEA). The purpose of this study was to assess the program’s effectiveness in achieving IDEA programming goals.

**Methods:**

Surveys were completed by youth (*n* = 342), caregivers (*n* = 2,375), and coaches (*n* = 1,406), and focus groups/interviews were conducted with 12 youth, 20 caregivers, and 9 coaches, diverse in race, ethnicity, ability, and other identities. Survey and focus group/interview questions addressed participants’ thoughts and experiences regarding inclusion, diversity, equity, andaccess in *Girls on the Run*.

**Results:**

Quantitative analyses of survey responses revealed favorable responses by all groups that the program: (a) provides a safe, inclusive, and supportive climate for all youth; (b) consists of teams with racially and ethnically diverse backgrounds; and, (c) successfully engages in strategies to reduce barriers to participation. Qualitative analyses of focus group/interview data resulted in 5 higher-order themes: (a) positive sentiments by girls, caregivers, and coaches; (b) social justice in the curriculum; (c) access to programming; (d) considerations regarding racial diversity; and, (e) serving gender-diverse participants.

**Discussion:**

Collective findings characterized *Girls on the Run* as successful in meeting its pledge toward inclusion, diversity, equity, and access to participation. All groups recognized the program’s positive impact on girls’ social and emotional learning and fostering an atmosphere of community connectedness. Curricular lessons and coach training align with evidence based strategies for inclusive and equitable programming, which can serve as an exemplar for other out-of-school-time programs.

## Introduction

Afterschool programs provide meaningful contexts for enhancing youths’ physical and psychosocial skills, health, and well-being (Mahoney et al., 2005; Smith, 2007; Pittman, 2017). In recent years, greater attention has been given to studying the characteristics, processes, and impact of out-of-school-time programs on PYD outcomes (Deutsch et al., 2017; Fredricks et al., 2017). Organized sports and physical activities connote a prevalent extracurricular pastime for children and adolescents (Weiss et al., 2012), offering opportunity for positive developmental outcomes such as close friendships, supportive relationships with adults, self-confidence, and motor skills enabling lifelong activity (Stuntz and Weiss, 2010; Eime et al., 2013; Goodway and Robinson, 2015). Attaining these benefits requires social, contextual, and cultural supports to ensure accessible and equitable growth and development for *all* youth (Leman et al., 2017; Simpkins et al., 2017; Smith et al., 2017).

Physical activity-based PYD (PA-PYD) programs in school and out-of-school-time settings are especially suited to bring about desirable psychosocial and behavioral outcomes (Weiss and Wiese-Bjornstal, 2009; Hellison, 2011; Weiss, 2011; Ward and Parker, 2013). PYD is a framework that highlights the strengths of young people by teaching attributes and skills to empower them to succeed and thrive in their everyday lives (Larson, 2000; Damon, 2004; Ramey and Rose-Krasnor, 2011; Moore, 2017). PYD programs consist of structured activities for optimizing social, emotional, and behavioral competencies (Eccles and Gootman, 2002; Roth and Brooks-Gunn, 2003). Contextual features include opportunities for learning skills that generalize beyond the target activity; an environment emphasizing safe, trusting, and supportive relationships; clear and consistent expectations; emphasis on autonomy and expression; encouragement of prosocial values and norms; a climate of inclusivity and belonging; and engaging interactions among family, school, and community. The PYD framework guides Girls on the Run’s curricula, coach training, and overall programming and, as such, formed the conceptual basis of the present study.

The PYD approach was framed by developmental psychologists with a focus on promoting academic, social, and emotional skills in school, home, and neighborhood contexts (Larson, 2000; Lerner et al., 2005; Benson, 2006). PYD was naturally appealing to sport science researchers and adapted for organized activities in school (e.g., physical education) and afterschool (e.g., sports) settings (Petitpas et al., 2005; Weiss and Wiese-Bjornstal, 2009; Hellison, 2011). While mainstream PYD and SEL researchers acknowledge sports and physical activities as *contexts* for youth development (Larson et al., 2006; Ramey and Rose-Krasnor, 2011; Jones et al., 2021), sport science researchers also identify *physical activity* level (frequency, intensity, duration), physical fitness, and fundamental motor skills as crucial behavioral competencies (Weiss and Wiese-Bjornstal, 2009; Weiss et al., 2012; Dzewaltowski and Rosenkranz, 2014). PA-PYD programs aim to improve both physical activity *and* social and emotional skills.

Enhancing physical activity alongside social and emotional learning is significant given the millions of youth in extracurricular pursuits but who still fall short of recommended activity levels (U.S. Department of Health and Human Services, 2018; Pfeiffer and Wierenga, 2019). Substantial evidence shows improved cognitive, psychosocial, and physical health for children and adolescents who engage in at least 60 min daily of moderate-to-vigorous physical activity (Donnelly et al., 2016; Hillman et al., 2017; U.S. Department of Health and Human Services, 2018). Only a small percentage of children and adolescents are meeting these guidelines (Designed to Move, 2012; Tremblay et al., 2016; U.S. Department of Health and Human Services, 2018), with percentages significantly lower for underserved and marginalized youth. Declining physical activity levels combined with increasing sedentary behaviors due to video gaming and social media (Banda and Robinson, 2017) reinforce the unique opportunity of PA-PYD programs to reverse this trend (Weiss et al., 2012; Dzewaltowski and Rosenkranz, 2014; Weiss, 2019).

The National Youth Sports Strategy (NYSS; U.S. Department of Health and Human Services, 2019) was implemented to motivate efforts by afterschool programs to meet physical activity guidelines, with special attention to underserved and marginalized populations—youth of color, youth of low-income families, youth living in rural communities, migrant youth, LGBTQ+ youth, and youth with disabilities. These populations have the most to gain from positive physical activity experiences but face many barriers to participation. NYSS highlighted *Girls on the Run* (GOTR) as an afterschool program dedicated to providing opportunities for *all* youth to improve physical activity and psychosocial outcomes. The current manuscript presents data of the program’s sustained efforts to offer, and assess effectiveness in delivering, inclusive, diverse, equitable, and accessible programming to enable participation benefits for *all* youth.

As a PA-PYD program, GOTR^1^ employs running, motor skills, and physical activities to develop 3rd to 8th grade girls’ social, emotional, *and* physical competencies for leading a healthy lifestyle (Riley and Britt, 2017). The mission, “we inspire girls to be joyful, healthy, and confident using a fun, experience-based curriculum which creatively integrates running,” is accompanied by a vision in which “every girl knows and activates her limitless potential and is free to boldly pursue her dreams.” The 10-week, 20-lesson intentional curriculum includes 75–90 min that *integrate* physical activity and social-emotional learning of developmentally-appropriate concepts: Identity (self-care and self-awareness), Connectedness (selecting and sustaining healthy relationships), and Empowerment (appreciating differences and celebrating strengths). The curriculum is designed to create space for participants’ experiences, cultures, and identities to shape the content of lessons, conversations, questions, and examples.

The curriculum aligns with Lerner’s *Five Cs* framework—confidence, competence, connection, caring, and character (Lerner et al., 2005; Lerner and Lerner, 2006). Lerner stated that a sixth C, contribution, is attainable after the *Five Cs* are demonstrated, and GOTR explicitly includes lessons on implementing a community impact project. Thus, the program aims to help girls develop social, emotional, and physical *competence*, feel *confident* in who they are, create positive *connections* with peers and adults, develop moral *character*, respond to others and oneself with *care* and compassion, and make a meaningful *contribution* to community. GOTR annually serves about 200,000 girls in ~175 Councils spanning all 50 United States, District of Columbia, and Canada. Evaluation research shows evidence of positive and sustained impact on social and emotional skills *and* physical activity levels (e.g., Bean et al., 2012; Ullrich-French and Cole, 2018; Weiss et al., 2019, 2020, 2021).

Safe, trusting, and supportive relationships are a hallmark of effective PYD programs, and GOTR maximizes this aspect through their national coach training. This is a requirement for new *and* returning coaches where they are actively engaged in learning strategies to deliver the curriculum with fidelity. The heart of this training revolves around the acronym BPM: Building Relationships; Positive, Inclusive Environment; and, Mastery Climate—key contextual features of effective PYD programs (Eccles and Gootman, 2002). Coaches are also prepared to deliver culturally responsive and inclusive lessons. During training, coaches reflect on how their social and cultural backgrounds, and those of girls on their teams, might affect attitudes and behaviors toward themselves and others. A module also covers social identities, bias, debiasing, and guiding conversations on social justice with their teams.

GOTR was recognized by Jones et al. (2017, 2021) in their comprehensive reports highlighting school and out-of-school-time programs meeting rigorous criteria for demonstrating positive impact on social and emotional learning with elementary-age youth. The program was identified as making predominant use of physical activities (labeled kinesthetic activities) as an instructional strategy for developing SEL competencies. Programs were evaluated on domains of cognitive skills (e.g., attention control), social skills (e.g., cooperative behavior), emotion skills (e.g., self-regulation), values (e.g., prosocial norms), perspectives (e.g., gratitude), and identity (e.g., self-esteem). GOTR was rated strongest in promoting SEL skills in social, identity, and values domains; having extensive support for community engagement; and demonstrating a commitment to equitable and inclusive education.

Since 2014, inclusion, diversity, equity, and access (IDEA) has been at the forefront of GOTR’s initiatives to elevate curricula, coach training, and programming. Readers are also directed to the inclusion and diversity link on the GOTR website. Jones et al. (2021) reported that GOTR strongly focuses on participation inclusivity, diversity, equity, and accessibility by integrating concepts into all aspects of program delivery. Examples include:

- providing scholarships to offset registration cost for low-income families;
- offering resources and materials in English and Spanish;
- partnering with the National Center on Health, Physical Activity and Disability (NCHPAD) to adapt lessons for girls with cognitive, sensory, and physical disabilities;
- training coaches on abuse prevention (in partnership with Darkness to Light), trauma-sensitive instruction, and disability inclusion;
- welcoming all girl-identifying youth as well as non-binary and gender-nonconforming youth; and
- framing lessons to counter gender stereotypes that negatively impact girls’ self-esteem, motivation, and health.

*Girls on the Run* sustains a commitment to inspire IDEA for *all* girls varying in race, ethnicity, culture, socioeconomic status, ability, and gender identity. In 2021, they made a pledge to advance their commitment in programming, coach training, and evaluation practices:

“We are committed to leveraging our intellectual, financial, and human resources to advance strategies to be inclusive, equitable and accessible to all. Our headquarters and councils are working to bring diverse voice to the table as we know that unique perspectives strengthen the quality and scope of our organization. We pledge to be a reflection of the communities we engage, not only in appearance, but also through fostering an atmosphere of community connectedness that serves as a model for our girls and community members.”

The overarching goal is that “all participants have a meaningful and engaging experience,” with objectives of updating programming and coach training; identifying strengths, opportunities, and areas for improvement; and, developing a sustainable system to advance IDEA priorities.

In recent years, PYD researchers place greater priority on conducting studies and evaluating program impact with diverse youth populations (Deutsch et al., 2017; Leman et al., 2017; Simpkins et al., 2017; Smith et al., 2017; Arellano et al., 2018). Simpkins and colleagues noted that Eccles and Gootman’s (2002) features of effective PYD programs defined universal developmental needs but may not be sensitive to youth varying in racial, ethnic, and cultural diversity. Efforts to review programming based on IDEA are needed to ensure that sociocultural norms and identities of diverse youth are addressed in participation experiences. As Simpkins (2015) argued, “It is time to bring equity, inclusion, and diversity to the forefront of research on organized activities. Issues related to equity, inclusion, and diversity need to be considered in terms of who has access to activities, the predictors of participation, how to design meaningful and effective activities, and the outcomes associated with activities” (p. 123).

Given the need for greater inclusion of diverse populations in evaluation efforts and GOTR’s pledge to elevate IDEA for girls, families, and coaches, the purpose of this study was to assess the program’s effectiveness in achieving IDEA goals in curricular offerings and coach training. The Mid-Atlantic Equity Consortium, Inc. (MAEC)^2^ was commissioned by GOTR to conduct an external review of initiatives to promote IDEA with multiple and diverse groups—youth participants, caregivers, and coaches. The ultimate goal of the evaluation was to build capacity for GOTR by identifying strengths and opportunities for sustaining IDEA in quality programming, coach training, and assessing impact. We conducted a secondary data analysis of the survey and focus group/interview data collected by MAEC; discuss how findings align with GOTR’s goals for sustaining an IDEA lens; and propose how study findings and related research can assist afterschool programs with strategies for prioritizing IDEA in programming consistent with PYD and SEL approaches. Based on studies evaluating the effectiveness of Girls on the Run in promoting socioemotional development (e.g., Ullrich-French and Cole, 2018; Weiss et al., 2020, 2021; Jones et al., 2021), and the program’s sustained commitment to a culturally responsive curriculum for *all* participants, we hypothesized that respondents of diverse identities will favorably appraise their experiences in the program as inclusive, equitable, and accessible.

## Method

### GOTR’s IDEA initiative defined

The overarching goal that “*all* participants have a meaningful and engaging experience” refers to people of all intersecting social identities—race, ethnicity, gender, ability, income level, religion, and others—are afforded opportunities to develop and improve social–emotional skills, cognitive abilities, and physical activity behaviors. To this point, each of the IDEA criteria are operationally defined and presented on the GOTR website:

- Inclusive: Girls on the Run is a place of belonging and welcomes, engages, and values all people.
- Diverse: The Girls on the Run movement mirrors the communities it serves. People of all races, ethnicities, thinking styles, abilities, generations, social roles, income levels, sexual orientation, gender identity, educational levels, and religions are represented and serve as active members of the organization.
- Equitable: Girls on the Run is a place where systemic disparities are acknowledged and addressed. Policies and practices ensure everyone can activate their limitless potential.
- Accessible: Everyone can fully participate in Girls on the Run programming, retrieve and utilize resources, and contribute through volunteer and employment opportunities.

### Advisory group

Prior to collecting data, a 23-member advisory group was convened to provide input about the content and relevance of survey and focus group/interview questions. Members included GOTR headquarters staff, Council staff, site liaisons, caregivers, former youth participants, and programming experts (e.g., youth development academics, NCHPAD coordinator). An initial meeting entailed GOTR and MAEC personnel introducing the project purposes and tasks requested on behalf of the advisory group. Follow-up communications involved feedback on survey and focus group/interview questions and initial findings.

### Survey measure

#### Composition

Surveys were developed in collaboration by MAEC evaluation team members and GOTR leadership staff to be administered via Qualtrics. Questions were intentionally designed to focus on participants’ thoughts and experiences regarding inclusion, diversity, equity, and access in Girls on the Run. Questions were tailored to youth participants, caregivers, and coaches, and available in English and Spanish. Many questions were asked in parallel format (e.g., “I feel safe at Girls on the Run”; “My child feels safe (socially, emotionally, and physically) at Girls on the Run,” “All participants on my team feel safe (socially, emotionally, and physically) at Girls on the Run”).^3^ Most questions included a 4-point Likert scale ranging from strongly disagree to strongly agree, with an option of “I do not know” (not included in analyses). Some items allowed for optional follow-up open-ended responses. Sample questions are seen in Table 1.

**Table 1:** Sample survey questions for youth, caregivers, and coaches.

Youth	Caregivers	Coaches
I have friends or teammates that I get along with at GOTR	My child has friends or teammates they get along with at GOTR	All participants on my team have friends or teammates they get along with at GOTR
I feel included in all activities at GOTR that I want to participate in	My child feels included and welcomed in all activities at GOTR that they want to participate in	All participants on my team feel included and welcomed in all activities they want to participate in
My GOTR coaches are about me	GOTR coaches care about my child and who they are as a person	All coaches on my team care about each participant and who they are as a person
I use what I learn during GOTR in my life (at home, in school, another sport, afterschool activity)	I feel that the content/topics my child learns about at GOTR are important for my child to learn	The GOTR lessons are relevant to all the participants on my team
My coaches listen to me when I talk with them	My child’s coaches communicate with me on a regular basis about what my child is learning in GOTR	The coaches on my team listen to participants when participants talk to them
My coaches take time to help or talk with me when I need it	My child’s coaches know how to relate to me when I talk with them	I know how to relate to participants on my team when I talk with them

Questions were rated on a 4-point Likert scale ranging from strongly disagree to strongly agree.

#### Participant numbers and demographics

Responses represented 76 Councils (of nearly 175), including youth participants (*n* = 342), caregivers (*n* = 2,375), and coaches (*n* = 1,406).^4^ Table 2 shows percentages by race/ethnicity for all groups. Less than 1% of youth were identified as nonbinary or transgender by their caregivers. A majority of the girls (64.7%) were in their first season in the program, with the remaining 35.3% indicating 2–6 seasons. For each season, time and intensity of exposure translates to 10 weeks, 2 sessions weekly, and 75–90 min per session, for a total of 20 lessons, 25–30 h, and a season-ending 5 K event.

**Table 2:** Race breakdown (%) for survey participants by group (*N* = 3,695).

Race	Youth (*n* = 286)	Caregivers (*n* = 2,132)	Coaches (*n* = 1,277)
American Indian/Alaskan Native	0	0	0.2
Asian	2.4	3.9	2.8
Black/African American	6.6	6.8	4.3
Hispanic/Latinx	6.3	6.1	3.8
Multiracial	10.5	8.4	3.5
Native Hawaiian/Pacific Islander	0	0	0.2
White/Caucasian	63.6	70.9	81.4
Other	2.8	0.1	0.7
Prefer Not to Say	7.7	3.8	3.1

Participants were coded as Multiracial if they selected more than one category (e.g., White and Native Hawaiian/Pacific Islander; Asian and American Indian/Alaskan Native).

Caregivers indicated being their child’s biological or birth parent (95%), with others a relative, legal guardian, or foster or adoptive parent. Coaches averaged 3 seasons’ experience in GOTR (*M* = 3.3, *SD* = 2.9), with 39% in their first season. Most coaches identified as female (97.4%); others identified as male (1.3%), nonbinary (0.08%), transgender (0.08%), or chose “prefer to self-describe” (0.16%) or “prefer not to say” (1.0%).

### Focus groups and interviews

#### Participants

A total of 41 individuals participated in 6 focus groups and 9 interviews, including 12 girls, 20 caregivers, and 9 coaches. There were 2 youth focus groups with 6 participants each, 2 caregiver focus groups with 5 and 6 individuals, and 2 coach focus groups with 3 and 4 individuals. Two coaches and 5 caregivers gave individual interviews, whereas 4 unrelated caregivers participated in paired interviews. Table 3 displays the racial breakdown by group.^5^

**Table 3:** Race breakdown for focus group/interview participants (*N* = 41).

Race	Youth (*n* = 12)	Caregivers (*n* = 20)	Coaches (*n* = 9)
Asian	2	0	0
Black/African American	5	8	0
Hispanic/Latinx	2	8	1
Native American	0	0	1
White/Caucasian	2	4	6
Other	0	0	1

Race was left blank for one youth participant.

#### Protocols

After drafting focus group and interview questions, MAEC sought input from the advisory group on wording, relevance, and cultural and linguistic sensitivity. All focus groups and interviews were conducted virtually by a facilitator and an assistant notetaker, including MAEC evaluation team or staff members. At least one person from the MAEC evaluation team was present at each focus group. All members of the MAEC evaluation team had at least a Master’s degree with graduate level coursework in qualitative methods. All had led focus groups for projects with other organizations prior to this external review. Interviewers and advisory group, evaluation team, and staff members represented diverse groups based on race, ethnicity, culture, nationality, gender, and education level.

For youth participants, the interviewer introduced herself and the session purpose to learn about their experiences in GOTR. Girls were apprised that responses would be confidential and they did not have to respond to questions that made them feel uncomfortable. The protocol was adapted from focus group guidelines with children (Terre des hommes, 2019). Sessions with caregivers and coaches also began with an introduction and purpose of learning about their child’s or team members’ experiences in GOTR, with a focus on whether and how the program is an inclusive, accepting, and equitable space for all youth participants.

For youth, questions centered on three issues: equity (referred to as fairness); being included, welcomed, and instilled with a sense of belonging; and, the curriculum. For caregivers, issues focused on equity; their child’s experiences; their own experiences and perceptions of the program; the curriculum; social justice; and, other considerations (e.g., inclusion of non-binary youth, diversity of friendships). For coaches, issues of equity, team members’ experiences, and the curriculum were also at the forefront, in addition to questions regarding coach training and parent/family engagement. Questions were followed with clarification and elaboration probes. Table 4 displays an abbreviated version of focus group/interview questions for all three groups.

**Table 4:** Abbreviated focus group/interview questions for all three groups.

Youth	Caregivers	Coaches
What does fairness look like and feel like in Girls on the Run?	What does equity look like and feel like in Girls on the Run?	What does equity look like and feel like in Girls on the Run?
Do you feel included and welcomed in the Girls on the Run activities that you want to participate in?	Does your child feel included and welcomed in all activities at Girls on the Run that they want to participate in?	As a coach, what have you done to ensure participants on your team feel included and welcomed in all activities at Girls on the Run that they want to participate in?
During Girls on the Run lessons, do you get to share stories or experiences about your own life?	From your perspective, how does Girls on the Run ensure that participants from diverse backgrounds feel that they belong?	How do you, as a coach, ensure participants from diverse backgrounds feel that they belong?
Do you feel that your coach(es) care about you and listen to you?	Do you believe coaches have positive relationships with participants, including participants from different socioeconomic backgrounds, religions, races, etc. Why or why not?	Have you experienced any challenges in developing connections with participants from diverse backgrounds, including participants from different socioeconomic backgrounds, religions, races, etc.? If so, can you elaborate?
Have you used anything you have learned during Girls on the Run in your life? (at home, in school, in another sport or afterschool activity? If so, how?)	From your perspective, what are critical issues related to equity that Girls on the Run should focus on in the lessons taught?	From your perspective, what are critical issues related to equity that Girls on the Run should focus on?

The full set of interview questions is available from the first author.

### Procedure/recruitment

GOTR’s Chief Program Officer contacted Council Directors about the study purpose and required tasks. Initially 87 Councils indicated interest, and additional information was provided about the survey, timeline, and recruitment strategies. This included an email restating the purpose (“gather the thoughts and experiences of GOTR participants, parents/guardians, and coaches related to inclusion, diversity, equity and access [IDEA] at GOTR”), templates for distributing to caregivers and coaches, and an online link and QR code to access the survey. Study participants were informed that responses were confidential and upon completion they would be eligible to win a gift card. At survey’s end, respondents were prompted about volunteering for a focus group or interview with an added gift card incentive. Volunteers provided demographic information, which experiences they could speak to (e.g., GOTR participants who are Black/African American; GOTR participants who identify as LGBTQIA+), and whether they permit their child to participate in a focus group or interview regarding their experiences. From this information, focus group and interview participants were identified using purposive sampling, and various affinity groups were formed to ensure diversity in race, ethnicity, language spoken at home, and types of GOTR experiences they could speak to. Surveys, focus groups, and interviews were conducted virtually.

### Data analyses

#### Quantitative analyses

We calculated means (*M*) and standard deviations (*SD*) for all survey items for girls, caregivers, and coaches. Independent *t*-tests were conducted to determine whether responses differed by race/ethnicity, comparing those who identified as White-only to those of any other racial or ethnic category, labeled as Black, Indigenous, People of Color (BIPOC).^6^ We applied a Bonferroni adjustment to protect against Type 1 errors (Tabachnick and Fidell, 2019). Statistical significance was set at *p* < 0.0031 for girls (0.05/16 items), *p* < 0.0026 for caregivers (0.05/19 items), and *p* < 0.0016 for coaches (0.05/32 items). Effect size (ES) was calculated using Cohen’s *d* (1988): *d* ≥ 0.20 = small, ≥0.50 = medium, ≥0.80 = large.

#### Qualitative analyses

Open-ended survey responses were coded to create a list of key topics and number of examples cited by respondents. Focus groups and interviews were recorded and transcribed verbatim. Facilitators and notetakers met to identify emergent themes within and across all group and individual sessions. Then, three members of the MAEC evaluation team read and deidentified the transcripts, assigning speaker codes and removing comments that could reveal identity. During this process, MAEC team members selected quotes that aligned with emergent themes identified in the group meeting and noted any additional themes. Then, one member of the MAEC team coded the deidentified transcripts in MAXQDA, a software program designed for computer-assisted qualitative and mixed methods data analysis, checking the document to ensure quotes aligned with themes and coding additional transcript segments. This team member then generated theme summaries, which were checked by facilitators, notetakers, and the MAEC evaluation team to ensure they accurately captured participants’ perspectives and experiences.

## Results

### Quantitative analyses: youth participants

Several survey items centered on feelings of inclusion in GOTR (e.g., “I am given the chance to talk about my own examples and things from my life …”; “I feel included in all activities … that I want to participate in”; “I can be myself at Girls on the Run”). A few items focused on positive developmental outcomes (e.g., “I have friends or teammates that I get along with at Girls on the Run”). For almost all items (14 of 16), responses tended toward “strongly agree” (>3.5), indicating high approval with their experiences of feeling safe, included, and treated equitably (Table 5). Ratings were also high for relevance of lesson content (e.g., “I use what I learn during Girls on the Run in my life [at home, in school, in another sport or afterschool activity]”). Five items related to perceptions about coaches, all receiving “strongly agree” ratings (e.g., “My coach cares about me”; “I feel comfortable talking to my coach”; “My coach takes time to help or talk with me when I need it”). No differences emerged for White and BIPOC participants on 15 of 16 items, the only exception (*p* > 0.0031; *d* = 0.31), “At Girls on the Run, everyone knows how to say my name or does their best to say my name the right way,” with White participants reporting stronger agreement (*M* = 3.73) than BIPOC participants (*M* = 3.55). However, both means indicate strong agreement and the effect size indicated small practical significance.

**Table 5:** Girls: means and standard deviations for all girls and for White and BIPOC groups, with effect size comparing race/ethnicity.

Item on girl survey	*M* (*SD*)			Cohen’s *d*
	All	White	BIPOC	
I enjoy Girls on the Run.	3.60 (0.58)	3.62 (0.51)	3.63 (0.58)	0.02
I am proud to be a part of Girls on the Run.	3.68 (0.52)	3.69 (0.48)	3.71 (0.51)	0.04
I have friends or teammates that I get along with at Girls on the Run.	3.69 (0.52)	3.70 (0.51)	3.70 (0.51)	0
I can be myself at Girls on the Run.	3.64 (0.56)	3.64 (0.53)	3.61 (0.62)	0.05
All of my teammates can be themselves at Girls on the Run.	3.66 (0.51)	3.66 (0.49)	3.63 (0.53)	0.06
At Girls on the Run, everyone knows how to say my name or does their best to say my name the right way.	3.67 (0.55)	3.73 (0.52)	3.55 (0.65)	0.31
I feel safe at Girls on the Run.	3.79 (0.42)	3.82 (0.39)	3.79 (0.46)	0.07
I feel included in all activities at Girls on the Run that I want to participate in.	3.70 (0.53)	3.73 (0.48)	3.73 (0.54)	0
I learn things at Girls on the Run that are interesting to me.	3.41 (0.64)	3.40 (0.60)	3.46 (0.59)	0.10
I use what I learn during Girls on the Run in my life (for example, at home, in school, in another sport or afterschool activity).	3.29 (0.66)	3.23 (0.62)	3.35 (0.67)	0.18
I am given the chance to talk about my own examples and things from my life at Girls on the Run.	3.54 (0.62)	3.58 (0.55)	3.54 (0.63)	0.07
My coaches provide me with choices and options.	3.66 (0.54)	3.66 (0.52)	3.74 (0.47)	0.16
My Girls on the Run coaches care about me.	3.79 (0.42)	3.78 (0.42)	3.83 (0.38)	0.13
My coaches take time to help or talk with me when I need it.	3.69 (0.51)	3.72 (0.46)	3.67 (0.52)	0.10
I feel comfortable talking to my coach(es).	3.63 (0.55)	3.66 (0.53)	3.59 (0.57)	0.13
My coach(es) listens to me when I talk with them.	3.74 (0.48)	3.77 (0.42)	3.75 (0.49)	0.04

N for the full girl sample was 342. Of these, 78 did not report race/ethnicity (either left blank or chose “prefer not to say”). Sample size for White was 181 and for BIPOC was 83.

### Quantitative analyses: caregivers

Responses reflected strong approval on items closely related to their child’s participation experiences reflecting inclusion and accessibility (e.g., “My child feels included and welcomed in all activities … they want to participate in”; “My child feels safe [socially, emotionally, and physically] at Girls on the Run”). They also felt strongly that lesson content was relevant and useful for their child. Caregivers affirmed that coaches cared about who their child was as a person. For those who reported their child required accommodations to participate, 100% agreed or strongly agreed that GOTR provided the accommodations needed (*M* = 3.72) and that their child was able to participate equitably to her peers (*M* = 3.76).

Caregivers strongly agreed GOTR is inclusive, accessible, and equitable for *themselves* (e.g., “My child’s coaches know how to relate to me when I talk with them”; “The coaches/staff at Girls on the Run verbally communicate with me in a way I can understand”). Scores for 2 items were just below 3.5: “My child’s coaches communicate with me on a regular basis about what my child is learning in Girls on the Run,” and “I am familiar with the Girls on the Run lesson content and its program goals.” These items had larger *SD’s* than other items, indicating greater variability in how familiar caregivers are with lessons.

Scores for 2 items were closer to 3.0: “It is important to me that the content presented during Girls on the Run lessons includes social justice topics,” and “The diversity of my child’s teammates is representative of the diversity in my community.” Again, the higher *SD’s* for these items indicate more varied perceptions. White and BIPOC caregivers differed (*p* < 0.0026) on these two items (Table 6); BIPOC agreed more strongly with both statements. Scores for both groups, however, were between “agree” and “strongly agree,” with effect sizes small for importance of including social justice topics (*d* = 0.44) and imperceptible for diversity of child’s teammates (*d* = 0.18). Perceived importance of teaching about social justice and whether their child’s team was representatively diverse were clarified in focus group/interview responses.

**Table 6:** Caregivers: means and standard deviations for all caregivers and for White and BIPOC groups, with effect size comparing race/ethnicity.

Item on caregiver survey	*M* (*SD*)			Cohen’s *d*
	All	White	BIPOC	
My child enjoys Girls on the Run.	3.73 (0.50)	3.73 (0.48)	3.79 (0.48)	0.13
My child is proud to be a part of Girls on the Run.	3.77 (0.47)	3.77 (0.44)	3.82 (0.44)	0.11
My child has friends or teammates they get along with at Girls on the Run.	3.74 (0.49)	3.76 (0.45)	3.73 (0.52)	0.06
My child can be their full self at Girls on the Run.	3.71 (0.51)	3.72 (0.49)	3.73 (0.51)	0.02
At Girls on the Run, everyone knows how to pronounce my child’s name or makes an effort to pronounce it correctly.	3.84 (0.39)	3.86 (0.36)	3.82 (0.43)	0.10
My child feels safe (socially, emotionally, and physically) at Girls on the Run.	3.81 (0.42)	3.82 (0.40)	3.82 (0.42)	0
My child feels included and welcomed in all activities at Girls on the Run that they want to participate in.	3.79 (0.44)	3.80 (0.43)	3.81 (0.43)	0.02
Girls on the Run coaches care about my child and who they are as a person.	3.82 (0.42)	3.83 (0.39)	3.83 (0.42)	0
My child’s coaches communicate with me on a regular basis about what my child is learning in Girls on the Run.	3.47 (0.76)	3.49 (0.74)	3.49 (0.76)	0
I am familiar with the Girls on the Run lesson content and its program goals.	3.38 (0.66)	3.37 (0.65)	3.46 (0.66)	0.14
I feel that what my child is learning at Girls on the Run is relevant to them and their life.	3.71 (0.48)	3.74 (0.45)	3.74 (0.46)	0
I feel that the content/topics my child learns about at Girls on the Run are important for my child to learn.	3.76 (0.44)	3.78 (0.42)	3.78 (0.43)	0
It is important to me that the content presented during Girls on the Run lessons includes social justice topics.	3.17 (0.86)	3.12 (0.85)	^*^3.46 (0.71)	0.44
The diversity of my child’s teammates is representative of the diversity in my community.	3.27 (0.71)	3.23 (0.70)	^*^3.36 (0.73)	0.18
My child’s coach(es) knows how to relate to me when I talk with them.	3.64 (0.54)	3.66 (0.52)	3.63 (0.55)	0.06
The coaches/staff at Girls on the Run verbally communicate with me in a way I can understand.	3.70 (0.50)	3.73 (0.48)	3.66 (0.55)	0.14
Girls on the Run’s written materials (e.g., flyers, emails, etc.) are provided in a language I can understand.	3.77 (0.44)	3.79 (0.42)	3.74 (0.47)	0.11
Girls on the Run provided the accommodations my child needed.	3.72 (0.45)	3.70 (0.47)	3.73 (0.46)	0.06
My child participated equally to her peers.	3.76 (0.43)	3.70 (0.47)	3.82 (0.40)	0.28

N for the full caregiver sample was 2,375. Of these, 306 did not report race/ethnicity (either left blank or chose “prefer not to say”). Sample size for White was 1,490 and for BIPOC was 579. For the last two items, only those who answered “Yes” to the question, “Does your child require any accommodations in order to fully participate in Girls on the Run?” responded (*n* = 50; 27 White, 22 BIPOC, 1 did not report race/ethnicity).

* Indicates significantly different from White (*p* < 0.0026).

### Quantitative analyses: coaches

Coaches favorably appraised the program’s accessibility, diversity, and inclusion and their role in reinforcing these ideals (Table 7). Coaches strongly believe they create a supportive and respectful environment, such as being familiar with team members’ cultural identities (e.g., race/ethnicity, languages spoken), knowing how to pronounce each participant’s name, and valuing individuals’ culture, identity, and who they are as a person. Similar to youth and caregivers, coaches highly rated GOTR experiences as safe, welcoming, and accommodating regardless of ability. They also strongly agreed that GOTR lessons were relevant and useful, and that examples and scenarios represented their team’s diversity.

**Table 7:** Coaches: means and standard deviations for all coaches and for White and BIPOC groups, with effect size comparing race/ethnicity.

Item on coach survey	*M* (*SD*)			Cohen’s *d*
	All	White	BIPOC	
All participants on my team enjoy Girls on the Run.	3.50 (0.58)	3.49 (0.57)	3.56 (0.59)	0.12
The participants on my team feel proud to be a part of Girls on the Run.	3.64 (0.50)	3.63 (0.50)	3.69 (0.48)	0.12
All participants on my team have friends or teammates they get along with at Girls on the Run.	3.61 (0.54)	3.62 (0.52)	3.60 (0.59)	0.04
All participants on my team feel they can be their full selves at Girls on the Run.	3.56 (0.55)	3.56 (0.54)	3.63 (0.55)	0.13
I am familiar with the cultural identities (e.g., race/ethnicity, languages spoken, religious affiliation, etc.) of each participant on my team.	3.43 (0.66)	3.43 (0.67)	3.51 (0.62)	0.12
I know how to pronounce the name of each participant on my team.	3.84 (0.39)	3.86 (0.37)	3.82 (0.40)	0.10
Each participant’s culture, identities, and who they are as a person are valued at Girls on the Run.	3.83 (0.39)	3.85 (0.37)	3.83 (0.41)	0.05
All participants on my team feel safe (socially, emotionally, and physically) at Girls on the Run.	3.76 (0.44)	3.76 (0.44)	3.82 (0.40)	0.14
All participants on my team can participate in all activities that they want to participate in, regardless of ability or disability.	3.81 (0.43)	3.83 (0.40)	3.82 (0.44)	0.02
All participants on my team feel included and welcomed in all activities that they want to participate in.	3.77 (0.44)	3.78 (0.43)	3.79 (0.45)	0.02
All coaches on my team care about each participant and who they are as a person.	3.87 (0.36)	3.89 (0.35)	3.85 (0.37)	0.11
The Girls on the Run lessons are relevant to all the participants on my team.	3.68 (0.53)	3.69 (0.51)	3.74 (0.51)	0.10
The examples and scenarios in the Girls on the Run lessons are representative of the diversity of participants on my team.	3.54 (0.59)	3.54 (0.58)	3.55 (0.61)	0.02
The skills and strategies participants learn through the Girls on the Run lessons are useful to them in their lives.	3.76 (0.44)	3.78 (0.42)	3.78 (0.44)	0
Each participant on my team is given the chance to talk about their own examples and experiences during the Girls on the Run practices.	3.82 (0.41)	3.83 (0.39)	3.81 (0.41)	0.05
It is important to me that the content presented during Girls on the Run lessons includes social justice topics.	3.33 (0.77)	3.31 (0.77)	*3.58 (0.66)	0.38
The diversity of the participants on my team is representative of the diversity of the Girls on the Run site (e.g., school, community center, etc.).	3.38 (0.68)	3.37 (0.68)	3.46 (0.69)	0.13
I provide choices and options to the participants on my team.	3.62 (0.50)	3.63 (0.50)	3.61 (0.51)	0.04
The coaches on my team listen to participants when participants talk to them.	3.83 (0.39)	3.85 (0.37)	3.82 (0.42)	0.08
I know how to relate to the participants on my team when I talk with them.	3.68 (0.48)	3.67 (0.48)	3.72 (0.48)	0.10
I feel equipped with the knowledge and skills to serve participants from diverse backgrounds including:
Race/ethnicity	3.52 (0.54)	3.49 (0.54)	^*^3.73 (0.47)	0.48
Socioeconomic status	3.55 (0.53)	3.53 (0.53)	^*^3.67 (0.49)	0.27
National origin	3.47 (0.56)	3.44 (0.55)	^*^3.65 (0.52)	0.39
English Learner status	3.38 (0.64)	3.32 (0.65)	^*^3.65 (0.54)	0.56
Gender identity/expression	3.37 (0.67)	3.35 (0.67)	^*^3.52 (0.61)	0.27
Religious affiliation	3.46 (0.56)	3.45 (0.55)	3.54 (0.55)	0.16
Disability status	3.49 (0.58)	3.47 (0.59)	3.59 (0.57)	0.21
My coach training prepared me to work with participants from diverse backgrounds.	3.30 (0.59)	3.31 (0.56)	3.36 (0.65)	0.08
Participants on my team have experienced barriers in accessing Girls on the Run (e.g., transportation challenges, physical accessibility, financial or language barriers, etc.)	2.16 (0.82)	2.15 (0.79)	2.20 (0.95)	0.06
I feel my local council provides the support and resources needed to address barriers participants/families may face in accessing Girls on the Run.	3.41 (0.57)	3.41 (0.56)	3.46 (0.54)	0.09
The coaches/staff at Girls on the Run communicate with parents/guardians in a way they can understand.	3.70 (0.47)	3.70 (0.48)	3.75 (0.45)	0.11
My council provides non-English translations of written materials (e.g., flyers, emails, etc.) to parents/guardians/family members who need it.**	3.40 (0.68)	3.39 (0.68)	3.50 (0.63)	0.17

N for the full coach sample was 1,406. Of these, 178 did not report race/ethnicity (either left blank or chose “prefer not to say”). Sample size for White was 1,030 and for BIPOC was 198. *Indicates significantly different from White (*p* < 0.0016). **About half of the coaches responded to the last item (*n* = 702), and the remainder either chose “I do not know” or left it blank; thus, the mean should be interpreted with this in mind.

Several questions prompted whether coaches felt knowledgeable and prepared to serve girls from diverse backgrounds. Scores ranged from 3.37 to 3.55 for feeling equipped with the knowledge and skills to serve various groups, with greater confidence reported for working with individuals diverse in race/ethnicity, socioeconomic status, and disability than for those of English Learner status and gender identity. Most coaches agreed their GOTR training prepared them to work with girls and families of diverse backgrounds and that their council provides resources to address barriers families may face when accessing GOTR.

Statistically significant differences emerged between White and BIPOC coaches for 6 items (*p* < 0.0016). First, BIPOC coaches felt stronger about the importance of including social justice topics in GOTR lessons. Second, BIPOC coaches felt better equipped to work with diverse participants based on 5 of 7 demographic groups: race/ethnicity, socioeconomic status, national origin, English Learner status, and gender identity. However, scores for both groups fell between “agree” and “strongly agree,” and effect sizes were mostly small (*d*’s = 0.27–0.48), with a medium effect size (*d* = 0.56) for English Learner status. No differences emerged for working with girls of varying religious affiliation and disability status.

### Qualitative analyses: open-ended survey questions

Two findings from open-ended responses were: (a) a majority of caregivers and coaches believe it is important to include social justice topics in lessons, and (b) a relatively small number of caregivers and coaches reported that girls experienced barriers in accessing GOTR. Caregivers (80.4%) and coaches (86.1%) agreed or strongly agreed with, “It is important to me that the content presented during Girls on the Run lessons includes social justice topics,” and were asked to describe topics that should be included (with examples of affirming diversity of all people, understanding bias, standing up against prejudice). Most follow-up responses included the 3 examples, along with several other topics (Table 8). One coach wrote, “I think that all topics of social justice should be taught or touched on. There was little diversity on the team, so teaching them to understand and confront bias and prejudice is important.”

**Table 8:** Percentage of caregivers and coaches who cited various social justice topics to be included in GOTR lessons, based on open-ended survey responses.

Topic	Caregiver % (*n* = 1,098)	Coach % (*n* = 793)
Understanding and affirming diversity	35.9%	37.2%
Understanding bias or implicit bias	20.1%	31.9%
Understanding and standing up against prejudice	26.2%	33.2%
Inclusion (including diversity and inclusion, being inclusive, etc.)	10.5%	5.7%
Understanding racism, being anti-racist, racial justice, race equity	8.3%	11.9%
Women’s rights/equality, gender equality, gender equity, gender stereotypes, understanding sexism, female empowerment	6.4%	3.2%
Standing up against bullying	4.5%	5.0%
LGBTQ+ needs/rights; gender identity and diversity	3.2%	3.9%
Ability, Disability needs/rights, special needs	1.7%	1.9%
Economic inclusion, diversity, justice; food insecurity	1.4%	1.7%

Only 3.6% of caregivers responded “yes” to, “My child has experienced barriers in accessing Girls on the Run (e.g., transportation challenges, physical accessibility, financial or language barriers).” Most followed by describing transportation, cost, and language barriers. One caregiver wrote, “… with the program ending at 4:50 it was a challenge to leave work. We relied on a lot of family help and having to leave work.” Location changes due to COVID-19 contributed to transportation issues because sessions were moved off-site due to no school programming. Some caregivers named cost as a barrier while others mentioned that cost was initially a barrier but they were able to receive aid: “We received financial support to participate. That was hugely helpful and much appreciated!”

For coaches, 27.4% agreed or strongly agreed that the girls on their team experienced barriers. When asked to describe, a majority listed similar barriers as caregivers, including transportation and cost. Coaches also reported location and scheduled times of GOTR as barriers and mentioned that some families started carpooling to address transportation challenges. Language translation was also cited as a barrier. One coach wrote, “Three of the seven girls [on my team] spoke Spanish at home. None of the coaches spoke Spanish. One email was translated by a GOTR director before the season started but there was no support after that.”

### Qualitative analyses: focus groups and interviews

Focus group/interview analyses within and across groups resulted in 5 higher-order themes: (a) positive sentiments by girls, caregivers, and coaches; (b) social justice in the curriculum; (c) access to GOTR programming; (d) considerations regarding racial diversity; and, (e) serving gender-diverse participants. Each theme is described, along with lower-order themes and example quotations. A visual of the higher-and lower-order themes is depicted in Table 9.

**Table 9:** Higher and lower-order themes from focus groups and interviews.

Higher-order theme	Lower-order theme
Positive sentiments by girls, caregivers, and coaches	Inviting, supportive space
	Inclusive curriculum
	Opportunities for learning
	Developing diverse friendships outside GOTR
Social justice in the curriculum	Mixed viewpoints on social justice
	Coach training and resources on social justice
Access to Girls on the Run programming	Scholarships are essential
	Planning for transportation
	Importance of language translation
Considerations regarding racial diversity	Diversity of GOTR participants
	Diversity of GOTR coaches
Serving gender diverse participants	Considerations regarding gender identity
	Coach training and resources on gender inclusivity

#### Theme 1: positive sentiments by girls, caregivers, and coaches (36 quotations)

Youth participants, caregivers, and coaches discussed many positive aspects of the program revolving around IDEA. Girls and caregivers believed coaches created a welcoming space. Girls discussed feeling supported, learning about interesting topics, and using strategies like Star Power in social contexts and situations outside GOTR (i.e., girls are taught to focus on their unique strengths to activate their power to shine). Coaches believe the curriculum fosters inclusivity, and caregivers talked about how their child’s unique experience as a GOTR participant has created opportunities for friendships outside the program.

The first lower-order theme is *Inviting, Supportive Space.* Caregivers and girls commented that coaches created a warm and friendly environment, provided individual attention, and encouraged girls to participate in a way most comfortable for them. A caregiver in the Latinx Affinity Group said (in Spanish), “In the group my daughter was the only Hispanic girl … considering [she] had just arrived in the United States and did not speak any English when she entered the program, she always felt very included. The coaches … made her feel welcome, happy … they did a great job in welcoming her and making her feel like she belong[ed].” One girl described, “So, we were doing the 5 K … there was this person on crutches, and she was so determined to finish … she did the whole 5 K with crutches on. The coaches … let her do it and they didn’t say that she was doing anything wrong. So, I felt that that was very equal … it was kind.” Other girls gave examples of encouragement: “They [coaches] give us like positive messages. Like if somebody said ‘I can’t do it’, they would say ‘you can do it’”; “At practices, my friends kept cheering me on to finish a lap.”

The second lower order theme is *Inclusive Curriculum.* Coaches emphasized that the curriculum is relevant and applicable to participants of all backgrounds. The focus on self-and other-acceptance fosters an inclusive environment. One coach expressed: “The curriculum really is inclusive in so many ways, beyond just language and race, but accepting who you are and loving who you are … and one of the things we loved was seeing the girls light up each other’s Star Power. Those girls could do that so quickly, find ways to compliment each other beyond just superficial … I think it’s a lesson that the girls actually take to heart.”

Girls commented on the third lower-order theme of *Opportunities for Learning*. When asked about the most interesting topics they learned during lessons, they mentioned appreciating individual differences, Star Power, being yourself, and inner beauty. One participant said, “… people can be different and still sometimes like the same things.” Another girl said, “I like the Star Power … because it was like you got to help others when they are kind of sad and then you helped them.” Girls also discussed using Star Power in domains outside GOTR, “The Star Power thing helped me in a lot of situations with my friends … [sometimes I would be asked for advice and] I would tell them some advice the Girls on the Run coaches taught me … because they were very clouded and I needed to bring the Star Power to life.”

As the fourth lower-order theme, caregivers noted that girls were *Developing Diverse Friendships Outside GOTR.* For example, the tee-shirt given to all GOTR participants helped girls meet friends outside of the program and fostered a connection. A caregiver in the Black/ African American Affinity Group gave this example: “I think [GOTR] promoted [cross-group friendships] without even knowing they actually were doing it … my daughter, when she got her shirt, she wore it to her school. And then some girls there saw it and they had a connection just because they had been in Girls on the Run before, even though they were not in the same program with her … it helped make friendships even outside of Girls on the Run.”

#### Theme 2: social justice in the curriculum (28 quotations)

Caregivers and coaches shared views about whether and how social justice topics should be included in the curriculum and coach training. The first lower-order theme is *Mixed Viewpoints on Social Justice.* Most participants advocated for including social justice topics while others questioned whether it would be appropriate or accepted. A caregiver in the Black/African American Affinity Group said, “It’s our reality right now. So, I think it’s appropriate and it could be tailored to their age … so they have a better understanding of what’s going on …” A caregiver in the Latinx Affinity Group said, “I think it’s always important. It helps them to become the leaders of tomorrow and show them there are certain things that we should … stand up for or we should not accept in life.”

A coach explained how social justice topics *build on* existing lessons, “I think part of Girls on the Run is getting girls to be comfortable in their own skin and empowering them. And then part of that is taking that empowerment and thinking about other groups of people and how they can use that for good … it’s just such an important thing to instill in this age group.” By contrast, some coaches felt that using the phrase “social justice” might turn some families away from GOTR. One coach said, “I feel like specific social justice topics … [are] just so riddled with politics and opinion [and] could be misinterpreted …” Other coaches acknowledged that social justice might be taught in ways to avoid offending some families by focusing lessons on compassion, respect, and standing up for others. One coach said, “I think it’s very important … to address [social justice topics] at Girls on the Run, but I am also on board with it through the lens of compassion, and understanding, and respect, because … it will just turn some families off immediately.” Some suggested that examples and scenarios already existing in the lessons could be updated to describe girls of various demographic backgrounds. One coach suggested this alternative scenario, “‘Your friend won the award that you really wanted. How would you treat her?’ Maybe instead it’s, ‘One of my friends called another one of my friends a racist name. How do you respond and what do you do?’”

Two respondents felt that social justice should be taught at home, not at GOTR. A caregiver in the Disability Affinity Group said, “I think there are definitely aspects [of social justice] that could be talked about but … there are certain things I want to have conversations with my children that I do not feel like anybody else should be talking to them about.” A coach *and* caregiver expressed: “… I’m not necessarily signing her [my child] up for an education in social justice. I’m signing her up to live in an experience that she’s going to learn about herself, and the people around her. And there’s going to be representation from a lot of different people on the team for her to learn about ….”

The second lower-order theme is *Coach Training and Resources on Social Justice.* A caregiver in the Latinx Affinity Group expressed the importance of training so that coaches “have the skills to steer the dialogue in the right way.” Coaches seemed keen on learning more about how to teach social justice topics and how to answer questions by the girls on their team, acknowledging that they do not feel completely prepared to discuss those topics. Some coaches suggested videos that model how coaches might respond to questions. They praised the safe space that GOTR creates for asking questions, so they anticipated that they will get questions about social justice topics. One coach explained, “Girls on the Run does such a great job of giving space for girls to talk about different things that are happening in life, happening in their school … if I felt equipped to discuss topics of social justice, then I feel like it could be done in a way that could be constructive.”

Caregivers and coaches appreciate that GOTR made updates to coach training over the years to include social justice topics. A caregiver in the Latinx Affinity Group said, “… Girls on the Run cares about the girls and cares about what they teach them. And how they want them to be involved within society, to love themselves more and to feel empowerment as a little girl … Because tomorrow … that girl could be our president.” A coach explained, “I would say that there was no diversity training whatsoever my first year … But as the world has changed and things evolve, those topics have been worked into the coach training … it’s good when you are working with an organization to hear that affirmation like, yes, we hear this is going on, we see you, we know the challenges you are going to face. This is how you may handle it.”

#### Theme 3: access to GOTR programming (24 quotations)

Some caregivers and coaches cited cost, transportation, and language as potential barriers to participating in GOTR and described strategies to help overcome them. The first lower-order theme is *Scholarships are Essential*. Respondents emphasized the importance of financial assistance for increasing access to the program. One coach stated, “… if it wasn’t for the scholarship program, then I think it would all be one economic background … The second team I coached was 100% funded by Girls on the Run. And then the other two were a mix, but I couldn’t tell the difference. All the girls seem very grateful to be there regardless of how they got there, but I know that there would be a good portion that couldn’t be there without funding.” A caregiver in the Latinx Affinity Group reinforced this point (in Spanish): “Girls on the Run granted my daughter a scholarship for a percentage of the program cost; As a single mother of three, the financial support allowed my daughter to have this experience; without it we would not have been able to have her participate.”

Some coaches explained that their councils put in extra effort to fundraise or form community partnerships to cover fees. One said, “We partnered with our community-based school team to make sure each girl got a new pair of running shoes for the season … we work with our community partners to make sure the girls are equipped to participate properly. Most of our girls are there on scholarship … because it is a pretty high rate of poverty in our school. So, having access to the program … these girls have a safe afterschool program to go to.” Another coach explained, “… our council does quite well, as far as making it accessible for anybody … we do unlimited scholarships, and we just fundraise more to cover that.”

The second lower-order theme is *Planning for Transportation.* Some respondents discussed that transportation challenges led to dropout or not being able to sign up in the first place. A caregiver in the Latinx Affinity Group described: “Unfortunately, there’s no sidewalks where we live. Let’s say we did not have a car. We couldn’t walk [to where we have practice] without jeopardizing life and limb. Equity would be making available transportation for those girls who needed it … it would be the difference between showing up and not showing up.” One coach elaborated, “transportation is always an issue, and it’s typically getting home in a school where it was 100% scholarship. Because a lot of parents work, and getting kids home at 5:00 at night can be a really big challenge … one girl ended up falling off a couple practices at the end of the season because she couldn’t get a ride home anymore.”

Some coaches arranged for transporting girls who needed it to participate. They identified the need early on, stayed late with a participant until their ride arrived, or provided transportation to and/or from practice.^7^ One coach said, “I worked really closely … with our community-based team on girls we knew might have a transportation issue, to identify that early and make sure that there was going to be transportation for these girls to get home.” One coach explained that prior to COVID-19 they would hold practice at school, so there was no need for transportation to practice. However, during COVID-19 restrictions, they were not allowed to hold practice at school sites which created a transportation barrier. This coach described, “[This last Fall and Spring there was] a single mom that didn’t drive. And if she didn’t have anybody to take her daughter, then her daughter, wasn’t going to be able to participate. So … I just said, ‘I’m happy to pick her up. I’m happy to take her home.’ Because I really wanted her to participate.”

The third lower order theme is *Importance of Language Translation.* Some caregivers and coaches said registration materials were available in English and Spanish, but noted they are often not translated to other languages in the community. Respondents also mentioned that while the GOTR website is a great resource, its reach is restricted due to limited language access. Some councils’ websites offer multiple languages, and some councils’ websites only offer English or both English and Spanish. One coach explained, “I know that there’s a family booklet we give out, and it can be in English or Spanish. But there’s so much great information on a website, but it’s only in English … I’ve always felt, both for international and the local councils, that it’d be great for families who … speak another language to be able to access that information ….”

One coach noticed that some girls would explain GOTR materials to their parents who do not speak English, so their council is trying to resolve this situation, “Our council’s working on translating materials like the application packet. Because a lot of our girls fill out the packet themselves and have their parents sign it. So, the parents do not really read through it.” One Spanish-speaking caregiver in the Latinx Affinity Group commented on their positive relationship with coaches in making the effort to understand each other. If there is coach turnover, however, this caregiver expressed the need to stay informed: “The teachers who were in charge were all Americans; none of them speak Spanish … If next season the coaches change, I will have to initiate communication … so that I am well connected/informed. Hopefully they are the same coaches, even though they do not speak my language … we try to understand one another … [laughs] They with me and I with them.”

#### Theme 4: considerations regarding racial diversity (18 quotations)

Most respondents felt their teams were diverse, but some discussed a noticeable lack of diversity. There were also mixed perceptions about diversity of the coaches. The first lower-order theme is *Diversity of GOTR Participants.* Most girls, caregivers, and coaches felt their teams were diverse in race, ethnicity, socioeconomic status, and language. A caregiver in the LGBTQIA+ Affinity Group described, “I think because it’s not with one school, “we’ve pulled from a really diverse group of kids … We see different colored faces and … the girls talk about that and they talk about their backgrounds. So, I think that’s been really a positive experience for my daughter.” Another caregiver in the Latinx Affinity Group said, “At the site I was at, every girl was treated pretty much the same. And my daughter was the only person who was not a Latina at this location. So, I think it was great that she got to be around people that are different from her, but she don’t have to feel any different at all. She made great friends, and all of the advisors treated them all well.”

A few respondents commented on the apparent lack of diversity on their teams, due to the makeup of their school or community composition. One caregiver in the Latinx Affinity Group said, “It would be very difficult for the teams to be diverse, because I’m pretty certain that the school is 95% White … There might be some Asians, some Latinos. Very few Black people. I think the Girls on the Run group probably reflects that … [The other families on the team] tend to be middle-class, White, highly educated.”

The second lower-order theme is *Diversity of GOTR Coaches*. One caregiver noticed and appreciated the coaching diversity on their daughter’s team, but others commented on lack of coach diversity and possible financial barriers to being a volunteer coach. A caregiver in the Asian or Multiracial Affinity Group said, “… there’s not a really big mix in ethnicities with the coaches … and probably because you have to be able to take time off of work to do that, and a lot of working parents that may add to that diversity may not be available to do so.” When asked for suggestions on how GOTR could recruit differently so that coaches are diverse and representative of their teams, a coach of color discussed the financial barrier due to GOTR being an unpaid volunteer experience: “In short of being able to pay coaches, I don’t know what else they could do, because I know it is a huge privilege for me to be able to have that much time that I can volunteer, and I know a lot of other women that do not have that privilege.”

#### Theme 5: serving gender diverse participants (8 quotations)

A few caregivers and coaches spoke to the need for more gender-inclusive language in the curriculum and coach resources, particularly on how to have conversations with participants and their families about gender identity. The first lower-order theme is *Considerations Regarding Gender Identity.* Some felt it is important to ask GOTR participants their pronouns and, along with adding “they” with he and she into the curriculum, this would help foster inclusivity. There were also questions about whether the title of the program is non-inclusive for those who are exploring gender identity. One caregiver in the LGBTQIA+ Affinity Group said, “I think it’s a little tricky when you’ve called yourself Girls on the Run, so that might be creating a hurdle. If you think about it from the gender perspective, with so many people now … being very fluid about their gender identity … several of my daughter’s friends are thinking about their gender identity right now, and they’re coming to Girls on the Run but they’re not necessarily identifying as a girl.” A coach who identified as queer shared concerns: “I was honestly pretty apprehensive because I don’t like how cis-normative it is … the name [Girls on the Run] makes it pretty non-inclusive to non-binary, queer, and/or trans youth. And there was never any conversation about pronouns or self-identified gender when doing introductions in camp … in the curriculum, there’s a lot of examples of ‘girl slash boy’, ‘him slash her’, where a gender-neutral term could very easily be [used] to further normalize that language for the [participants].”

The second lower-order theme is *Coach Training and Resources on Gender Inclusivity.* Coaches desire training specific to gender identity, both in terms of their own education as well as recommendations for how to talk to participants in an age-appropriate way. One coach said, “… it would be important for coaches to have the resources so that they can, on a personal level, understand the actual things they would be talking about before trying to teach about them. For example, if there was a lesson about gender identity, I think it’d be pretty hard to teach that without prior knowledge and understanding of sex versus gender and the gender spectrum.” Another expressed: “… if Girls on the Run changes their perspective [on gender] or expands the way they speak about participants … as a coach I would want some training on how to navigate that with other coaches, parents, and the girls at the level of awareness they are at … talking about this to a third to fifth grader would be much different than talking to a parent. I would want to know what’s the recommended language, because just thinking about it makes me cringe … because I can think about all of those questions ….”

## Discussion

*Girls on the Run* is an evidence-based PA-PYD program, offering access and equity for diverse and underserved youth and demonstrating efficacy in promoting social, emotional, and behavioral competencies (Jones et al., 2017, 2021; U.S. Department of Health and Human Services, 2019; Weiss et al., 2019, 2020, 2021). The program’s mission, vision, and core values embrace an IDEA lens and the organization engages in continuous efforts to revise programming to sustain that commitment. The purpose of the present study was to conduct a large-scale evaluation of the effectiveness with which GOTR is achieving IDEA objectives in their curricular content and delivery. This was accomplished by employing mixed methods with multiple, diverse groups to determine perceptions of curricular offerings, girls’ participation experiences, and coach training. In the following paragraphs, we summarize key findings and implications and connect them with literature on strategies for designing equitable and inclusive activities for diverse youth.

Survey responses revealed highly favorable impressions from youth participants, caregivers, and coaches regarding GOTR’s efforts to offer accessible, inclusive, and equitable opportunities for girls from diverse backgrounds. In fact, *all* items were scored above “agree” with the majority “strongly agree” for the quality and relevance of learning experiences that reflect an IDEA lens. That is, the practice environment is characterized as a safe, welcoming, and inclusive space, and activities are designed in a culturally responsive way for girls and families of all racial and ethnic backgrounds. Girls engage in valuable opportunities for learning behavioral and social–emotional skills that generalize beyond the program, such as ability to form diverse friendships in varied social contexts. Caregivers and coaches alike agreed that girls are valued for who they are as a person regardless of cultural background as well as (dis)ability or English Learner status. Positive perceptions prevailed among White *and* BIPOC groups regarding programming from an IDEA lens.

Focus groups and interviews added detailed narrative to clarify, explain, and interpret the high survey ratings. Positive sentiments predominated discussions among various affinity groups, supporting and elaborating upon survey findings that GOTR is characterized as an inviting, supportive space; the lessons are inclusive and culturally responsive; opportunities for learning social–emotional skills abound; and identifying as a GOTR participant lends opportunities for forming friendships with diverse peers. The social justice theme revealed that coaches and caregivers value lessons on compassion, respect, and standing up for others, and they praised GOTR for creating a safe space for participants to ask questions. Coaches appreciated GOTR’s continued efforts to update training related to social justice, but they also desired additional training and resources. Access to programming despite potential barriers was deemed successful due to scholarship funding, options for resolving transportation challenges, and efforts to translate communications to families. Collectively, quantitative and qualitative findings revealed opportunities for skill building, a sense of belonging, and supportive relationships, among other features, that align with evidence-based best practices for promoting PYD among diverse youth (Deutsch et al., 2017; Simpkins et al., 2017; Smith et al., 2017; Jones et al., 2021).

Although the majority of qualitative responses supported favorable survey ratings, some caregivers and coaches voiced alternative perspectives about social justice in the curriculum, access to programming, and gender diversity. Though fewer in number, their expressions are valuable and provide insights for reinforcing GOTR’s pledge to “bring diverse voice to the table as we know that unique perspectives strengthen the quality and scope of our organization.” Interviewees frequently affirmed the importance of including social justice in the lessons, but contrary views raised the potential for “turning families off” or that values should be “taught at home, not at GOTR.” A large percentage of caregivers (81%) and coaches (86%) agreed or strongly agreed with, “importance of including social justice content in the lessons,” but greater variability of opinions existed on this topic than others. It should be noted that respondents were not asked whether social justice topics are *already* included in the curriculum, which some are (e.g., standing up for others, embracing differences, empathy, and community impact project). Rather, caregivers were asked, “how important is it to you” that GOTR content includes social justice topics. Thus, caregivers may not be aware of existing lessons on social justice and, indeed, findings indicated that many are not familiar with the lessons their children experience. Thus, more dialogue is needed among GOTR administrators, families, and coaches to share what is included in curriculum content and how to deliver it in an age-appropriate way.

Multiple viewpoints about what IDEA content is taught to elementary-age youth pose an important opportunity to sustain GOTR’s pledge of ensuring a culturally responsive approach to designing activities for *all* youth. A keystone of effective PYD programs is “integration of family, school, and community efforts” (Eccles and Gootman, 2002; Simpkins et al., 2017), so making curricular decisions along an IDEA lens will benefit from candid discussions and collaborations among GOTR personnel, caregivers, coaches (many who are school teachers), and community leaders. The social-ecological model (Bronfenbrenner and Morris, 1998) and developmental systems models (Sameroff, 2010; Lerner, 2017) highlight the *interactive influence* of significant others (e.g., caregivers, teachers, coaches) and social contexts (e.g., schools, organized activities, cultural norms) on children’s acquisition of beliefs, attitudes, and behaviors. Developing youths’ moral values toward social justice, such as standing up against discrimination, bias, and prejudice, will be achieved through a concerted effort by respected individuals in their social system. GOTR is strategically positioned, alongside family, school, and community, to influence morals and values that respect racial, ethnic, and cultural diversity.

Coaches expressed confidence in GOTR’s comprehensive training for preparing them with the knowledge and skills to work with participants of diverse backgrounds. Focus groups and interviews revealed a desire for additional training on strategies to teach social justice topics and answer questions in an age-appropriate manner. GOTR is continually updating coach training to include relevant content and effective methods that engage coaches in problem-solving and decision-making. Training modules include social issues and scenarios inclusive of girls with diverse backgrounds. Coaches are required to complete the entire training covering consistent lesson delivery, disability inclusion, and trauma-informed instruction. A challenge for GOTR has been ensuring that coaches fulfill all of these modules prior to the season. Findings prompt further interest in training experiences that include content, activities, and methods for addressing topics in a culturally and developmentally-appropriate way.

BIPOC coaches reported greater ability to work with girls of diverse backgrounds (small effect size), an important revelation given that over 80% of coach survey respondents were White. Predominance of White coaches naturally raises the need for recruiting coaches who are racially, ethnically, and culturally diverse. Youth are more likely to identify with models who are similar in characteristics such as race, ethnicity, and culture, which results in greater motivation to learn and adopt behaviors and skills being taught (Weiss and Wiese-Bjornstal, 2009). Respondents did not readily provide strategies for attracting more diverse coaches, so this is another area of collaboration among GOTR personnel, families, and community. This has been and continues to be an organizational priority. Interestingly, some participants paired lack of coach diversity with inability to volunteer due to work commitments, suggesting that diverse coach recruitment may also be related to social class disparities.

Survey responses suggested access to and equity in participation, for example through scholarships, partnering with NCHPAD to enable accommodations, and communications in English and Spanish. Caregivers strongly agreed that their child felt included in all activities and participated equally to peers. Coaches praised their council for providing resources to offset barriers families might face in accessing GOTR. In focus groups and interviews, which featured greater participant diversity, some caregivers and coaches expressed concerns that disadvantaged families faced financial, transportation, and language barriers. They voiced a need to further reduce disparities by providing more scholarships, fundraising opportunities, transportation options, and translation to languages other than just English and Spanish. These barriers to accessing participation—financial, transportation, and language—are common for out-of-school-time programs and especially affect families of color, lower income, and migrant status (Simpkins et al., 2017; Smith et al., 2017).

While focus groups/interviews mostly revolved around access and inclusion based on racial, cultural, and socioeconomic diversity, “serving *gender diverse* participants” emerged as a theme among a smaller but vocal group of coaches and caregivers. Some recommended more gender-inclusive language in the curriculum and the need for more training and resources to effectively teach lessons and answer youths’ questions in an age-appropriate way. GOTR welcomes youth who are non-binary or gender-nonconforming and want to participate in a girl-centered program. Thus, the program is inclusive of diverse gender identities, but this information may not be known or noticed by families. GOTR can explore ways to make the Grown-Up Guide (resource for caregivers) more accessible and reinforce the importance of caregivers investing time to engage with their child on social issues, which again invokes the importance of youth learning from multiple individuals in their social system.

PYD and SEL researchers highlight challenges and strategies for designing culturally responsive activities (Whitley et al., 2015; Forneris et al., 2016; Deutsch et al., 2017; Simpkins et al., 2017; Jones et al., 2021). Simpkins et al. offered a framework for designing activities to meet the needs of racially-and ethnically-diverse youth by mapping strategies onto the eight contextual features of effective PYD programs (Eccles and Gootman, 2002). They argue that scarce attention has been given to cultural competence as a life skill, and afterschool activities are prime contexts for assisting racially and ethnically diverse youth in attaining skills such as resolving cultural differences and exploring identities. Their framework and strategies offer a roadmap for GOTR and other programs to optimize equitable participation for diverse youth, especially underserved and marginalized youth who have the most to gain from a program delivered by caring and supportive adults who provide appropriate structure, positive norms, and support for autonomy and belonging (Simpkins et al., 2017; Smith et al., 2017).

Self-selection is one of the limitations of the study. Councils chose whether to participate in the study and respondents voluntarily opted into completing the survey and/or participating in a focus group or interview. Thus, favorable survey ratings and focus group/interview responses may be elevated as a result of the voluntary nature of participation and not having potentially differing opinions by those who chose not to participate. Second, we were able to capture many aspects of diversity such as race, ethnicity, ability, socioeconomic status, language, and gender identity, which are regarded *visible* forms of diversity. A limitation is that we were unable to assess aspects of *invisible* diversity, such as ways of thinking, learning, processing, communicating, and behaving (i.e., neurodiversity). Future research evaluating PA-PYD program impact might consider ways to assess both visible and invisible forms of diversity. Finally, GOTR programming transitioned to offering three delivery modes from Spring 2020 to Spring 2021: 100% virtual, 100% in-person, and hybrid. These variations may have affected how girls, caregivers, and coaches perceived experiences, although Fall 2020 season findings showed that all modes were received favorably by caregivers and coaches and open-ended narrative revealed evidence of season-long impact (Weiss et al., 2021). Due to these limitations—participant self-selection, not assessing invisible forms of diversity, and variations in program delivery—the results of this study may not generalize to other populations and programs.

In conclusion, collective findings from quantitative and qualitative data characterized GOTR as being successful in meeting the pledge of inclusion, diversity, equity, and access to participation. All groups provided information that recognizes GOTR’s positive impact on girls’ social and emotional learning; they also provided diverse voices and varied perspectives needed for fulfilling GOTR’s promise of “… fostering an atmosphere of community connectedness that serves as a model for our girls and community members.” GOTR lessons and coach training align with evidence-based strategies for inclusive and equitable programming, which can serve as an exemplar for other out-of-school-time programs. Varied opinions on social justice issues such as racial, socioeconomic, and gender inclusivity provide GOTR with additional areas of opportunity for sustaining their commitment to providing a culturally responsive space for *all* youth and achieving goals of promoting children’s healthy behaviors and life skills now and in the future.

## Data availability statement

The data analyzed in this study are subject to the following licenses/restrictions: the datasets presented in this article are not readily available because they are proprietary to Girls on the Run International. Requests about the data should be directed to AR, ariley@girlsontherun.org.

## Ethics statement

The project was reviewed by Research Integrity and Compliance (RIC), Texas State University, San Marcos, TX, USA. Because the study exclusively involved the examination of anonymous, secondary data, the research is not regulated by RIC, and written informed consent for participation was not required.

## Author contributions

MW and LK drafted the early versions of the manuscript, analyzed the quantitative data, and interpreted the qualitative themes. AR provided the secondary data set. All authors conceived the study, contributed to revising the manuscript, and approved the final submitted version.

## Conflict of interest

The authors declare that the research was conducted in the absence of any commercial or financial relationships that could be construed as a potential conflict of interest.

## Publisher’s note

All claims expressed in this article are solely those of the authors and do not necessarily represent those of their affiliated organizations, or those of the publisher, the editors and the reviewers. Any product that may be evaluated in this article, or claim that may be made by its manufacturer, is not guaranteed or endorsed by the publisher.

## Footnotes

^1^ www.girlsontherun.org

^2^MAEC was founded in 1992 as an education non-profit dedicated to increasing access to high-quality education for culturally, linguistically, and economically diverse learners.

^3^Caregivers were asked to collaborate with and help their daughter do the survey. Caregivers who were also coaches completed the survey twice, once for each perspective.

^4^≥90% of the respondents identified Girls on the Run (grades 3–5) as their program experience; others named Heart & Sole (grades 6–8) and Camp GOTR (shortened summer program).

^5^For focus groups and interviews, the caregiver’s race served as a proxy for their child’s race because the volunteer sign-up at the end of the survey only asked for the family member’s and not their child’s race.

^6^Sample sizes for racial groups within BIPOC (e.g., Hispanic, Asian, Native Hawaiian/Pacific Islander) were relatively small and disproportionate compared to White, so we used the BIPOC category in our analyses for more stable estimates.

^7^According to GOTR policy, coaches can only provide transportation if the caregiver authorizes the coach on their registration paperwork.
